# Machine learning-based predictive model for hungry bone syndrome following parathyroidectomy in secondary hyperparathyroidism

**DOI:** 10.3389/fendo.2025.1635451

**Published:** 2025-09-05

**Authors:** Yalin Chai, Nan Yuan, Jiaming Yin, Bing Shen, Lijie Sun, Lin Zhang, Le Yin, Xuan Wang, Feng Luo, Congjuan Luo

**Affiliations:** The Affiliated Hospital of Qingdao University, Qingdao, China

**Keywords:** hungry bone syndrome, secondary hyperparathyroidism, parathyroidectomy, risk factors, machine learning

## Abstract

**Objective:**

To develop an interpretable machine learning model for predicting hungry bone syndrome (HBS) risk following parathyroidectomy in secondary hyperparathyroidism (SHPT) patients.

**Methods:**

This retrospective study analyzed 181 SHPT patients who underwent parathyroidectomy at the Affiliated Hospital of Qingdao University (2015 - 2025). Participants were randomly divided into a training group (70%) and a validation group (30%). From 46 candidate variables, five key predictors were selected through logistic regression and Boruta algorithm. Seven machine learning models were trained, evaluated by ROC curves, calibration curves, and decision curve analysis (DCA). Model interpretability was quantified via SHapley Additive exPlanations (SHAP).

**Results:**

The XGBoost algorithm demonstrated excellent predictive performance, with an AUC of 0.878 (95% CI: 0.779 - 0.973) and an F1 score of 0.871 for the validation cohort. The key predictors included preoperative parathyroid hormone (Pre-PTH), the percentage decay between Pre-PTH and PTH at skin closure (%PTH), alkaline phosphatase, serum calcium, and age. Additionally, we designed a web application to estimate HBS risk.

**Conclusions:**

This interpretable machine-learning model is effective in predicting the risk of HBS in SHPT patients after parathyroidectomy, thereby providing guidance for postoperative surveillance strategies.

## 1 Introduction

Secondary hyperparathyroidism (SHPT), a prevalent complication of chronic kidney disease (CKD), contributes to bone lesions, vascular calcification, and elevated risks of cardiovascular events and mortality (1–3). Epidemiological studies indicate that SHPT affects 20%-80% of CKD patients, with prevalence rates correlating with disease severity and dialysis duration (4). Current therapeutic strategies encompass vitamin D analogs (e.g., calcitriol) (5), calcimimetics (e.g., cinacalcet), and phosphate binders (6). For refractory cases, parathyroidectomy (PTX) remains the definitive intervention per KDIGO guidelines, particularly in CKD patients with persistent hyperparathyroidism (intact parathyroid hormone [iPTH] >800 pg/mL) (7, 8). PTX has been shown to improve survival rates by 15%-57% in dialysis-dependent patients (9) and alleviate symptoms such as pruritus, bone pain, and fracture risk (10–13).

Postoperative hungry bone syndrome (HBS), a complication affecting 25%-75% of PTX cases (14–16), is clinically characterized by prolonged hypocalcemia (corrected serum calcium <2.1 mmol/L for >4 days) (17, 18). The pathophysiology involves accelerated bone remodeling under chronic PTH stimulation, followed by abrupt mineralization and calcium influx into osteoid tissue after rapid postoperative PTH decline, resulting in severe hypocalcemia (19, 20). Elevated preoperative PTH levels are strongly associated with HBS development (21–23). Notably, recent studies propose that %PTH (the percentage decay between Pre-PTH and PTH at skin closure) predicts hypocalcemia following thyroidectomy (24). However, this metric has not yet been evaluated as a predictor of HBS in SHPT patients undergoing PTX. We investigated the relationship between %PTH and postoperative HBS and used machine learning to develop a predictive model for the occurrence of HBS after parathyroidectomy in SHPT patients.

## 2 Methods

### 2.1 Patients and designs

This retrospective cohort study enrolled patients diagnosed with secondary hyperparathyroidism who underwent parathyroidectomy at the Affiliated Hospital of Qingdao University between January 2015 and May 2025. Inclusion criteria required comprehensive preoperative clinical evaluation and documented serum calcium measurements within 72 hours postoperatively. The study protocol was reviewed and approved by the Ethics Committee of the Affiliated Hospital of Qingdao University (Approval No. QYFY WZLL 29980). Sample size determination adhered to the 10 events per variable (EPV) criterion, ensuring that the minimum number of outcomes for binary classifications exceeded 10-fold the number of independent variables in the predictive model.

**Figure 1:**
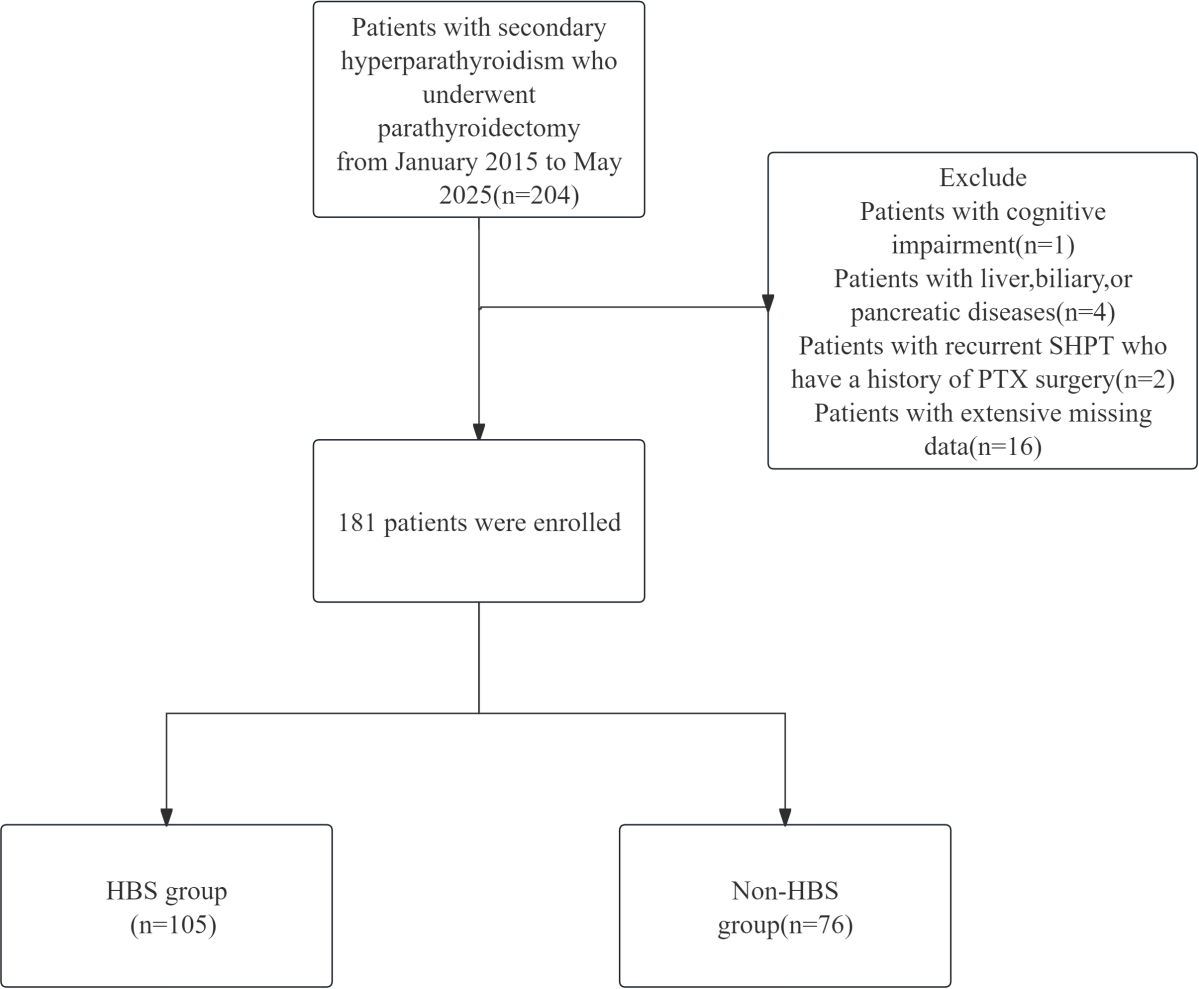
The patient flow chart in our study.

Surgical indications for parathyroidectomy were defined as meeting at least one of the following criteria:(1) Severe SHPT (persistent intact parathyroid hormone [iPTH] >800 pg/mL) refractory to pharmacological therapy with calcitriol or vitamin D analogs;(2)Severe SHPT accompanied by hyperphosphatemia (serum phosphate >2.0 mmol/L);(3)Severe SHPT with clinically significant symptoms (intractable pruritus and/or bone pain);(4)Radiologically confirmed parathyroid hyperplasia (maximum gland diameter >1.0 cm).Exclusion criteria comprised:(1)Concurrent hepatobiliary or pancreatic disorders;(2)Cognitive impairment impairing informed consent or follow-up compliance;(3)Recurrent SHPT with prior parathyroidectomy history. The flow chart of the study is shown in **Figure 1**.

### 2.2 Data collection and definitions

Demographic and clinical characteristics were systematically collected, including: General characteristics: Sex, age, height, body mass index (BMI), dialysis modality (hemodialysis/peritoneal dialysis), dialysis vintage, and clinical manifestations (bone pain, height reduction, pruritus); Preoperative management: Use of calcimimetics (Cinacalcet), vitamin D analogs (Calcitriol, Paricalcitol, Alfacalcitol), phosphate binders(Lanthanum carbonate), and medication for osteoporosis (Bisphosphonates); Laboratory parameters: Pre-PTH, PTH at skin closure, calcium, potassium, phosphate, albumin, alkaline phosphatase (ALP), cystatin C, creatinine, urea, uric acid, triglycerides, total cholesterol, low-density lipoprotein (LDL), high-density lipoprotein (HDL), and hemoglobin; Surgery-related factors: Procedure type, number of excised parathyroid glands; Comorbidities:hypertension, diabetes mellitus, coronary artery disease, cerebrovascular disease, osteoporosis, fracture.

Blood samples were obtained within 72 hours postoperatively. Patients were stratified into two groups based on corrected serum calcium levels:HBS group: Corrected calcium <2.1 mmol/L; Non-HBS group: Corrected calcium ≥2.1 mmol/L. Corrected calcium formula: Corrected Ca (mmol/L) = ionized Ca + (40 − serum albumin [g/L]) × 0.02.The percentage decay between Pre-PTH and PTH at skin closure(%PTH): [(Preoperative PTH – PTH at skin closure)/Preoperative PTH] × 100%. Blood was collected uniformly 20 minutes after total thyroidectomy (before skin closure) to control half-life effects.

### 2.3 Calcium management

Heparin-free dialysis was implemented during the final preoperative session and the initial postoperative session. Postoperative calcium monitoring followed a tiered protocol. The average medication dosage for managing hypocalcemia after parathyroidectomy varies among patients, but typically involves a combination of intravenous and oral calcium supplements. The recommended dose of intravenous calcium gluconate is 2 mg/kg/h, with the median total dose reported in studies being approximately 8.2g (Interquartile Range 6.1 - 10.3g). Oral calcium supplements usually consist of 3 - 6g elemental calcium daily (7.5 - 15g calcium carbonate), which should be combined with active vitamin D (e.g., calcitriol 0.5 - 2.0 μg/day). Discharge criteria included: Stable corrected calcium within normal range; Absence of hypocalcemia-related symptoms; No surgery-associated complications.

### 2.4 Statistical analysis and model development

All statistical analyses were conducted using R software (version 4.3.1, R Foundation for Statistical Computing, Austria), with a significance level set at P < 0.05.The normality test and the chi-square test were used for the measurement data, and the normally distributed data were expressed as mean ± standard deviation, and the t-test was used for comparison between groups; the non-normally distributed data were expressed as median (interquartile spacing), and the Mann-Whitney U-test was used for comparison between groups; the counting data were expressed as percentage (%), and the χ2-test or Fisher’s exact probability method was used for comparison between groups. ‘s exact probability method. MissForest (random forest interpolation technique) was used to interpolate the data, and data with more than 20% missing were discarded. The training set and test set are separated and interpolated respectively to avoid information leakage.

Use LASSO regression, Boruta method and logistic regression to select the final feature variables used in the model. In logistic analysis, variables with p < 0.05 are considered as potential risk factors. The LASSO method selects features and reduces dimension by narrowing the coefficients, retaining the features with large contributions and eliminating redundant features. Boruta is a feature selection method that determines the importance of each variable by comparing its Z-score. Three methods were selected to select the common characteristic variables as the final variables of the model. This method improves the accuracy of the model, reduces the risk of overfitting and excludes irrelevant predictors. Machine learning is more advantageous for classification and prediction and can handle features more efficiently than traditional statistical methods (22, 25, 26). Data were trained on the following seven ML models: Logistic Regression (LR), Adaptive Boosting (AdaBoost), Support Vector Machine (SVM), eXtreme Gradient Boosting (XGBoost), Categorical Boosting (CatBoost), K-Nearest Neighbors (KNN), and Neural Network (NN). We used grid search to optimize the parameters. The performance of the prediction model was evaluated by the AUC of the ROC curve, calibration curve, specificity, accuracy, recall, F1 score. Decision curve analysis (DCA) was plotted to assess clinical utility. For the most performance-driven diagnostic models, we revalidated their generalization capabilities using 10-fold cross-validation to prevent overfitting. The SHAP feature importance ranking and SHAP beeplot visually demonstrate each feature’s contribution to the final prediction, while the SHAP plot intuitively visualizes how different features influence individual predictions.

## 3 Results

### 3.1 Characteristics of the participants

From January 2015 to May 2025, 181 eligible secondary hyperparathyroidism (SHPT) patients undergoing parathyroidectomy (PTX) were included in the final analysis. These patients were randomly allocated into a training cohort (n=128) and a validation cohort (n=53) at a 7:3 ratio. Among the 181 participants, 105 (58%) developed postoperative hungry bone syndrome (HBS). The overall missing data situation is shown in **Supplementary Table 1**. Compared with the non-HBS group, patients in the HBS group had a significantly lower median age (P <0.001). Regarding comorbidities, the HBS group showed higher prevalence of osteoporosis (P = 0.017). In terms of preoperative medication use, the HBS group demonstrated higher usage rates of paricalciferol (P = 0.037) and bisphosphonates (P = 0.040). For preoperative symptoms, the HBS group exhibited higher proportions of osteodynia, eight reduction, and pruritus. These findings are detailed in **Table 1**. In terms of surgical methods, the proportion of SPTX was lower in HBS group (P <0.05), and the proportion of TPTX and TPTX+AT was higher. The proportion of HBS group with ≥4 parathyroid glands removed was higher, and the proportion of <4 parathyroid glands removed was lower. The results are shown in **Table 2**. Laboratory data comparison (**Table 3**) showed that compared with the non-HBS group, the HBS group had a higher percentage of Ca, Bun, Pre-PTH, PTH at skin closure, %PTH than the non-HBS group (P <0.05).

**Table 1:** Comparison of General characteristics in the Non-HBS and HBS groups.

Variable	Levels	Overall (N = 181)	Group		P-value
			Non-HBS (N = 76)	HBS (N = 105)	
General characteristics					
Age(years)		46.65 (10.27)	50.84 (9.46)	43.62 (9.80)	<0.001
Gender, n (p%)	male	92.00 (50.83%)	38.00 (50.00%)	54.00 (51.43%)	0.850
	female	89.00 (49.17%)	38.00 (50.00%)	51.00 (48.57%)	
H(cm)		165.01 (8.42)	164.74 (7.49)	165.20 (9.07)	0.708
Wt(kg)		64.23 (13.35)	65.11 (13.31)	63.59 (13.41)	0.449
BMI(kg/m2)		24.51 (13.33)	24.15 (4.41)	24.77 (17.13)	0.725
Dialysis time(years)		7.35 (3.16)	7.82 (3.65)	7.01 (2.71)	0.106
Dialysis modality, n (p%)	Hemodialysis	168.00 (92.82%)	70.00 (92.11%)	98.00 (93.33%)	0.752
	Peritoneal dialysis	13.00 (7.18%)	6.00 (7.89%)	7.00 (6.67%)	
Cinacalcet, n (p%)	no	139.00 (76.80%)	57.00 (75.00%)	82.00 (78.10%)	0.626
	yes	42.00 (23.20%)	19.00 (25.00%)	23.00 (21.90%)	
Calcitriol, n (p%)	no	80.00 (44.20%)	31.00 (40.79%)	49.00 (46.67%)	0.432
	yes	101.00 (55.80%)	45.00 (59.21%)	56.00 (53.33%)	
Paricalcitol, n (p%)	no	175.00 (96.69%)	71.00 (93.42%)	104.00 (99.05%)	0.037
	yes	6.00 (3.31%)	5.00 (6.58%)	1.00 (0.95%)	
Alfacalcidol, n (p%)	no	174.00 (96.13%)	71.00 (93.42%)	103.00 (98.10%)	0.107
	yes	7.00 (3.87%)	5.00 (6.58%)	2.00 (1.90%)	
Lanthanum carbonate, n (p%)	no	167.00 (92.27%)	72.00 (94.74%)	95.00 (90.48%)	0.290
	yes	14.00 (7.73%)	4.00 (5.26%)	10.00 (9.52%)	
Bisphosphonates, n (p%)	no	178.00 (98.34%)	73.00 (96.05%)	105.00 (100.00%)	0.040
	yes	3.00 (1.66%)	3.00 (3.95%)	0.00 (0.00%)	
Height reduction, n (p%)	no	167.00 (92.27%)	73.00 (96.05%)	94.00 (89.52%)	0.105
	yes	14.00 (7.73%)	3.00 (3.95%)	11.00 (10.48%)	
Osteodynia, n (p%)	no	95.00 (52.49%)	43.00 (56.58%)	52.00 (49.52%)	0.348
	yes	86.00 (47.51%)	33.00 (43.42%)	53.00 (50.48%)	
Pruritus, n (p%)	no	136.00 (75.14%)	55.00 (72.37%)	81.00 (77.14%)	0.463
	yes	45.00 (24.86%)	21.00 (27.63%)	24.00 (22.86%)	
Hypertension, n (p%)	no	45.00 (24.86%)	23.00 (30.26%)	22.00 (20.95%)	0.153
	yes	136.00 (75.14%)	53.00 (69.74%)	83.00 (79.05%)	
Coro2ry heart disease, n (p%)	no	145.00 (80.11%)	58.00 (76.32%)	87.00 (82.86%)	0.277
	yes	36.00 (19.89%)	18.00 (23.68%)	18.00 (17.14%)	
Diabetes, n (p%)	no	170.00 (93.92%)	72.00 (94.74%)	98.00 (93.33%)	0.696
	yes	11.00 (6.08%)	4.00 (5.26%)	7.00 (6.67%)	
Cerebrovascular disease, n (p%)	no	168.00 (92.82%)	71.00 (93.42%)	97.00 (92.38%)	0.789
	yes	13.00 (7.18%)	5.00 (6.58%)	8.00 (7.62%)	
Osteoporosis, n (p%)	no	177.00 (97.79%)	72.00 (98.63%)	105.00 (100.00%)	0.017
	yes	4.00 (2.21%)	1.00 (5.26%)	3.00 (0.00%)	
Fracture, n (p%)	no	177.00 (97.79%)	73.00 (96.05%)	104.00 (99.05%)	0.176
	yes	4.00 (2.21%)	2.00 (3.95%)	2.00 (0.95%)	

**Table 2:** Comparison of Surgery-related factors in the Non-HBS and HBS groups.

Variable	Levels	Overall (N = 181)	Group		P-value
			Non-HBS (N = 76)	HBS (N = 105)	
Surgery-related factors					
Surgical time(minutes)		137.32 (56.47)	132.13 (56.66)	141.08 (56.30)	0.295
Number of resections	1	1.00 (0.57%)	1.00 (1.35%)	0.00 (0.00%)	0.064
	2	12.00 (6.86%)	8.00 (10.81%)	4.00 (3.96%)	
	3	20.00 (8.57%)	11.00 (12.16%)	9.00 (5.94%)	
	4	148.00 (84.00%)	56.00 (75.68%)	92.00 (90.10%)	
TPTX, n (p%)	no	147.00 (81.22%)	65.00 (85.53%)	82.00 (78.10%)	0.206
	yes	34.00 (18.78%)	11.00 (14.47%)	23.00 (21.90%)	
SPTX, n (p%)	no	150.00 (82.87%)	58.00 (76.32%)	92.00 (87.62%)	0.046
	yes	31.00 (17.13%)	18.00 (23.68%)	13.00 (12.38%)	
TPTX+AT, n (p%)	no	64.00 (35.36%)	29.00 (38.16%)	35.00 (33.33%)	0.503
	yes	117.00 (64.64%)	47.00 (61.84%)	70.00 (66.67%)	

**Table 3:** Comparison of Biochemical indicators in the Non-HBS and HBS groups.

Variable	Overall(N = 171)	Group		P-value
		Non-HBS (N = 76)	HBS (N = 105)	
Biochemical indicators				
Hb(g/L)	108.66 (16.89)	110.57 (15.28)	107.27 (17.91)	0.183
WBC (109/L)	5.75 (1.67)	5.77 (1.82)	5.73 (1.56)	0.886
CRP(mg/L)	4.87 (5.66)	4.66 (5.35)	5.02 (5.90)	0.670
Ca (mmol/L)	2.43 (0.20)	2.50 (0.21)	2.39 (0.18)	<0.001
K(mmol/L)	5.01 (0.74)	4.90 (0.75)	5.09 (0.72)	0.084
P(mmol/L)	2.24 (0.54)	2.20 (0.56)	2.27 (0.53)	0.405
Pre-PTH(pg/ml)	1,633.45 (1,000.47)	976.45 (482.33)	2,108.99 (1,010.39)	<0.001
PTH at skin closure(pg/ml)	198.22 (125.36)	174.60 (122.91)	215.31 (124.92)	0.030
%PTH	84.55 (12.18)	79.39 (15.63)	88.29 (6.86)	<0.001
Scr(µmol/L)	807.96 (258.24)	801.67 (244.34)	812.52 (268.91)	0.778
Bun(mmol/L)	22.66 (6.82)	21.31 (6.65)	23.64 (6.80)	0.023
CYSC(mg/L)	6.74 (1.71)	6.47 (1.69)	6.93 (1.72)	0.076
UA(µmol/L)	362.50 (119.60)	361.71 (119.58)	363.08 (120.18)	0.939
ALP(U/L)	354.19 (410.73)	161.39 (169.91)	493.73 (473.64)	<0.001
ALB(g/L)	39.69 (4.44)	40.22 (4.88)	39.30 (4.08)	0.185
TG(mmol/L)	1.66 (0.92)	1.81 (1.14)	1.56 (0.71)	0.091
TC(mmol/L)	4.31 (1.17)	4.16 (1.27)	4.41 (1.08)	0.170
HDL(mmol/L)	2.45 (0.69)	2.37 (0.71)	2.50 (0.67)	0.187
LDL(mmol/L)	1.18 (0.39)	1.13 (0.34)	1.21 (0.42)	0.136

### 3.2 Feature selection

**Figure 2:**
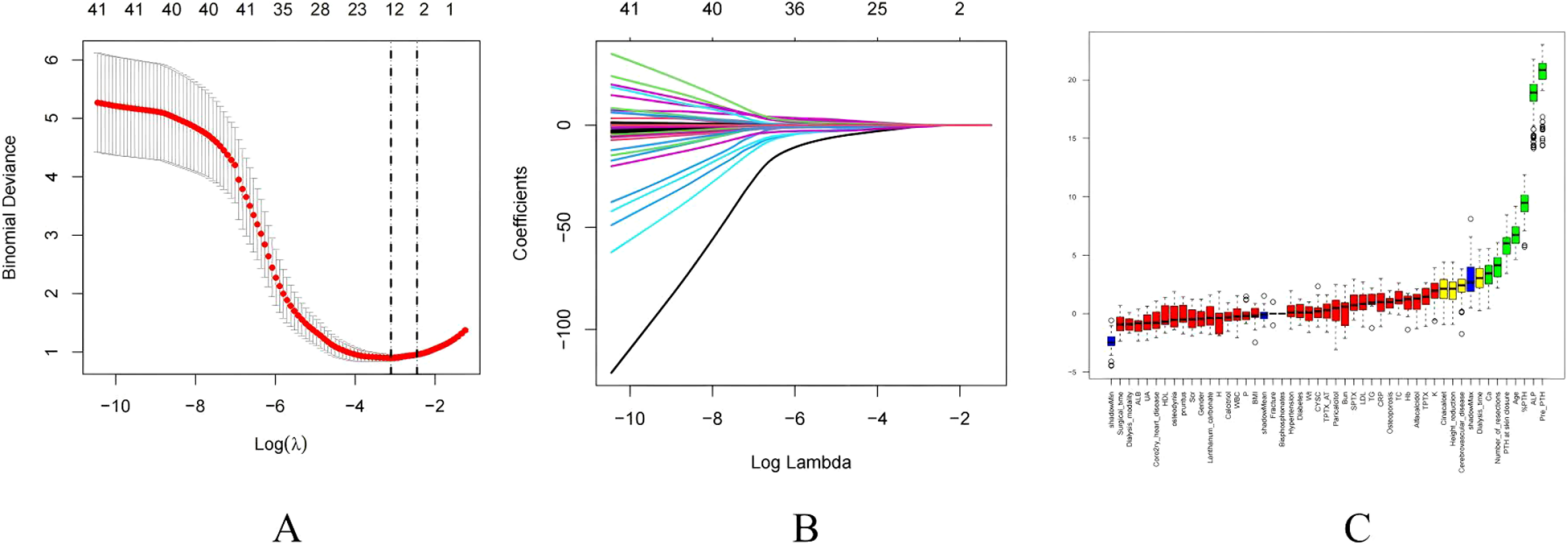
Feature selection. **(A)** The relationship between Lambda (regularization parameter) and CVM (mean cross validation error) in Lasso regression; **(B)** Lasso regression Lambda and Coefficients Plot. **(C)** Feature selection based on Boruta principle. Green boxes indicate important variables, red boxes indicate unimportant variables.

The selection of variables was based on the overlapping results of logistic regression, Lasso regression and Boruta’s algorithm. Univariate analysis showed (**Table 4**) that %PTH had a p-value <0.001, indicating that %PTH was significantly associated with postoperative HBS. In addition, Pre-PTH (p<0.001)), age (p<0.001), Ca (p=0.014), ALP (p<0.001), TPTX (p=0.045), SPTX (p=0.029) and the number of parathyroid glands resected (p=0.004) were significantly different between the HBS and non-HBS groups. All the above significant parameters were included in the multifactorial logistic regression analysis. In the LASSO regression analysis, variable coefficients are shown in **Figure 2A**, while the relationship between regularization parameter (λ) and mean cross-validation error (CVM) is illustrated in **Figure 2B**. The five variables identified through LASSO regression as strongly associated with HBS include Pre-PTH, ALP, %PTH, Age, and Ca. The regression coefficients for these variables are detailed in **Supplementary Table 2**. The Boruta method identified variables including Pre-PTH, ALP,%PTH, Age, PTH at skin closure, number of parathyroid glands removed, and Ca (**Figure 2C**). Ultimately, these five variables—Pre-PTH, ALP, %PTH, Age, and Ca—were selected for subsequent analyses.

**Table 4:** Univariate and multivariate logistic regression analysis.

Variable	Levels	OR (univariable)	OR (multivariable)
Age(years)		0.96 (0.92 - 0.99, p=.026)	0.96 (0.91 - 1.01, p=.152)
Gender, n (p%)	male		
	female	1.22 (0.61 - 2.47, p=.572)	
H(cm)		0.99 (0.95 - 1.03, p=.570)	
Wt(kg)		0.99 (0.96 - 1.01, p=.270)	
BMI(kg/m2)		1.01 (0.98 - 1.04, p=.620)	
Dialysis time(years)		0.90 (0.80 - 1.00, p=.056)	
Dialysis modality, n (p%)	Hemodialysis		
	Peritoneal dialysis	1.23 (0.28 - 5.39, p=.782)	
Cinacalcet, n (p%)	no		
	yes	1.41 (0.59 - 3.37, p=.434)	
Calcitriol, n (p%)	no		
	yes	0.77 (0.38 - 1.58, p=.480)	
Paricalcitol, n (p%)	no		
	yes	0.17 (0.02 - 1.58, p=.119)	
Alfacalcidol, n (p%)	no		
	yes	0.27 (0.05 - 1.46, p=.129)	
Lanthanum carbonate, n (p%)	no		
	yes	2.29 (0.44 - 11.83, p=.321)	
Bisphosphonates, n (p%)	no		
	yes	0.00 (0.00-Inf, p=.987)	
Hypertension, n (p%)	no		
	yes	2.16 (0.94 - 4.94, p=.070)	
Coro2ry heart disease, n (p%)	no		
	yes	0.80 (0.35 - 1.86, p=.608)	
Diabetes, n (p%)	no		
	yes	0.71 (0.17 - 2.99, p=.645)	
Cerebrovascular disease, n (p%)	no		
	yes	12614529.59 (0.00-Inf, p=.986)	
Osteoporosis, n (p%)	no		
	yes	0.00 (0.00-Inf, p=.988)	
Fracture, n (p%)	no		
	yes	0.00 (0.00-Inf, p=.988)	
Surgical time(minutes)		1.00 (1.00 - 1.01, p=.523)	
Number of resections		3.18 (1.44 - 7.00, p=.004)	1.55 (0.38 - 6.23, p=.540)
TPTX, n (p%)		2.76 (1.02 - 7.48, p=.045)	2.60 (0.61 - 11.02, p=.194)
SPTX, n (p%)		0.31 (0.11 - 0.88, p=.029)	1.46 (0.17 - 12.90, p=.732)
TPTX+AT, n (p%)		0.98 (0.47 - 2.06, p=.958)	
Hb(g/L)		0.99 (0.96 - 1.01, p=.176)	
WBC (109/L)		0.90 (0.73 - 1.12, p=.352)	
CRP(mg/L)		1.04 (0.97 - 1.12, p=.280)	
Ca (mmol/L)		0.10 (0.02 - 0.63, p=.014)	0.06 (0.00 - 1.15, p=.062)
K(mmol/L)		1.43 (0.86 - 2.40, p=.171)	
P(mmol/L)		1.00 (0.53 - 1.90, p=.990)	
Pre-PTH(pg/ml)		1.00 (1.00 - 1.00, p<.001)	1.00 (1.00 - 1.00, p=.003)
PTH at skin closure(pg/ml)		1.00 (1.00 - 1.01, p=.185)	
%PTH		1.10 (1.04 - 1.15, p<.001)	1.03 (0.97 - 1.09, p=.357)
Scr(µmol/L)		1.00 (1.00 - 1.00, p=.935)	
Bun(mmol/L)		1.04 (0.99 - 1.10, p=.143)	
CYSC(mg/L)		1.09 (0.89 - 1.34, p=.419)	
UA(µmol/L)		1.00 (1.00 - 1.00, p=.844)	
ALP(U/L)		1.01 (1.00 - 1.01, p<.001)	1.00 (1.00 - 1.01, p=.151)
ALB(g/L)		0.96 (0.89 - 1.04, p=.287)	
TG(mmol/L)		0.80 (0.53 - 1.21, p=.290)	
TC(mmol/L)		1.36 (0.98 - 1.88, p=.067)	
HDL(mmol/L)		2.11 (0.77 - 5.73, p=.144)	
LDL(mmol/L)		1.59 (0.93 - 2.70, p=.088)	

### 3.3 Performance comparison of different ML methods

Following feature selection, seven machine learning algorithms—Logistic Regression (LR), Adaptive Boosting (AdaBoost), Support Vector Machine (SVM), eXtreme Gradient Boosting (XGBoost), Categorical Boosting (CatBoost), K-Nearest Neighbors (KNN), and Neural Network (NN)—were trained to predict postoperative HBS.

To compare the need to include %PTH, we compared the difference in AUC values between whether or not to exclude %PTH. The results showed that the AUC value of the model including %PTH was higher (**Supplementary Table 3**), so we included %PTH for further analysis.

**Figure 3:**
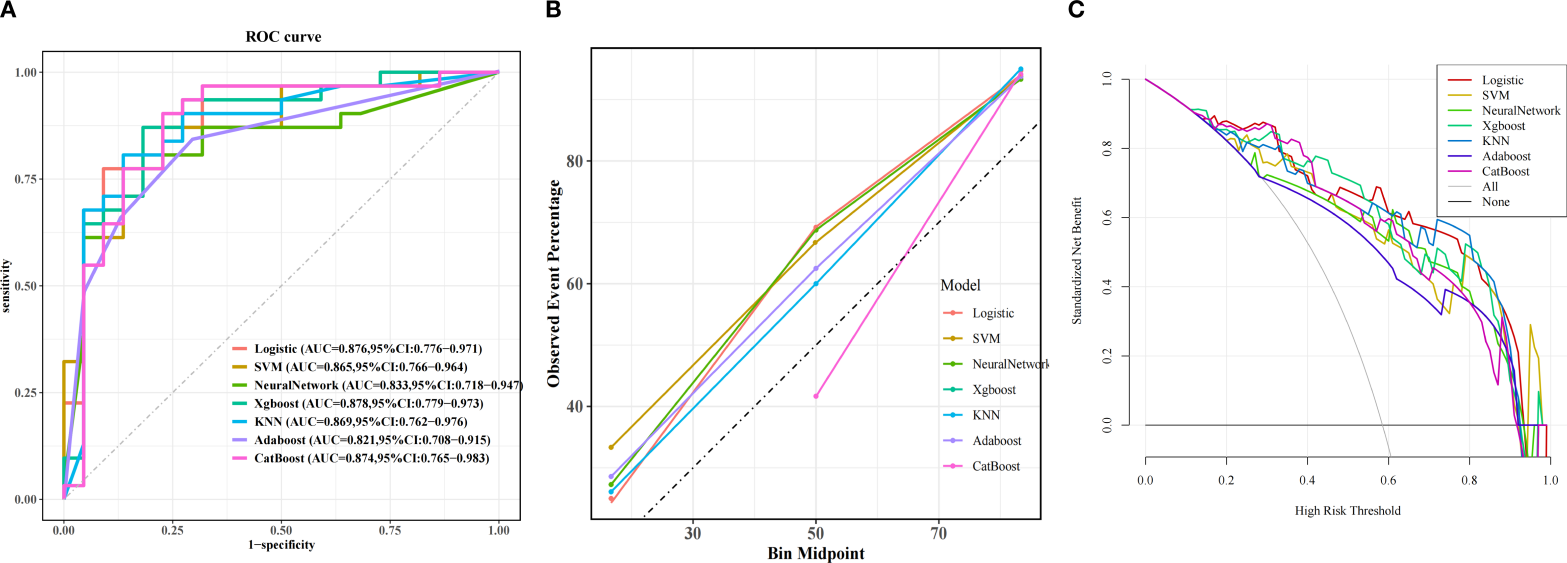
Predictive performance of the model in validation cohorts. **(A)** Receiver Operating Characteristic (ROC) curves for the validation cohorts. The curves illustrate the discriminatory ability of different predictive models, with the area under the ROC curve (AUC) values displayed for each model: Logistic Regression (LR), eXtreme Gradient Boosting (XGBoost), Adaptive Boosting (AdaBoost), Support Vector Machine (SVM), K-Nearest Neighbor (KNN), Gradient Boosting Machine (GBM), Categorical Boosting (CatBoost). **(B)** Calibration Curve. Exhibited excellent alignment between predicted probabilities and observed outcomes. **(C)** Decision Curve Analysis (DCA). The curves show the net benefit of each model across various threshold probabilities.

Among machine learning models, the XGBoost model (AUC = 0.878) showed the best performance, followed by LR (AUC = 0.876), CatBoost (AUC = 0.874), KNN (AUC = 0.869), SVM (AUC = 0.865), NN (AUC = 0.833) and AdaBoost (AUC = 0.821). (**Figure 3A**). Classification in the validation cohort outperformed all models with an F1 score of 0.871 and an accuracy of 0.849. Detailed performance metrics for all models, including specificity, precision, and accuracy, are summarized in **Table 5**. The calibration curves show a very good agreement between predicted probabilities and observations (**Figure 3B**). In addition, decision curve analysis (DCA) confirmed its optimal net benefit across clinically relevant probability thresholds (**Figure 3C**).

**Table 5:** Detailed performance metrics for all models in validation cohorts.

Model	Accuracy	Sensitivity	Specificity	F1
Logistic	0.83 (0.654 - 0.923)	0.774 (0.518 - 0.968)	0.909 (0.738 - 1.000)	0.842 (0.648 - 0.933)
SVM	0.83 (0.672 - 0.923)	0.871 (0.679 - 1.000)	0.773 (0.500 - 1.000)	0.857 (0.713 - 0.944)
NeuralNetwork	0.811 (0.662 - 0.912)	0.774 (0.645 - 1.000)	0.864 (0.500 - 1.000)	0.828 (0.695 - 0.926)
Xgboost	0.849 (0.654 - 0.923)	0.871 (0.518 - 0.972)	0.818 (0.750 - 1.000)	0.871 (0.678 - 0.948)
KNN	0.83 (0.711 - 0.962)	0.806 (0.603 - 1.000)	0.864 (0.750 - 1.000)	0.847 (0.674 - 0.932)
Adaboost	0.755 (0.672 - 0.923)	0.806 (0.580 - 1.000)	0.682 (0.577 - 1.000)	0.794 (0.657 - 0.913)
CatBoost	0.849 (0.769 - 0.982)	0.903 (0.720 - 1.000)	0.773 (0.625 - 1.000)	0.865 (0.711 - 0.948)

**Figure 4:**
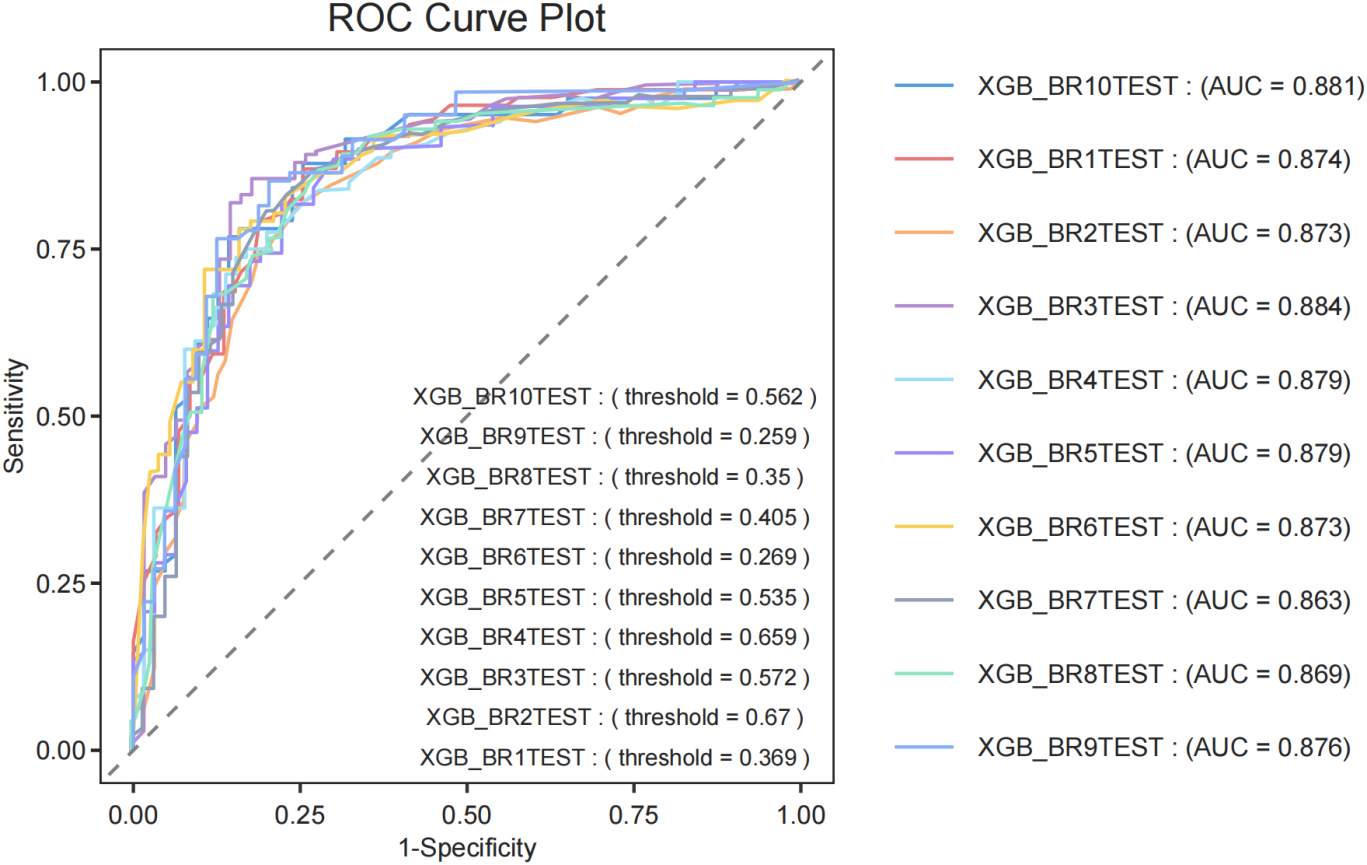
Ten fold cross validation evaluation. ROC curve plot of XGBoost.

To evaluate the generalization capability of the top-performing diagnostic models, we conducted cross-validation with 10-fold resampling to prevent overfitting. The ROC curve results (**Figure 4**) confirm that our constructed model demonstrates excellent generalization performance without signs of overfitting or underfitting. Based on these findings, the XGBoost model was identified as the most effective model in this study.

### 3.4 Clinical decision threshold analysis

ROC curve analysis with the AUC-ROC index identified a statistically optimal threshold of 0.489 (95% CI: 0.483 - 0.509), achieving 87.1% sensitivity and 81.8% specificity at this level (**Supplementary Table 3**). The decision curve demonstrated a net benefit of 0.3614 at the 35% threshold, confirming its clinical applicability. We established a three-tier risk stratification system: Low-risk (<20%): Standard calcium supplementation regimen; Moderate-risk (20 - 35%): Close monitoring; High-risk (≥35%): Intensive calcium supplementation regimen. The XGBoost prediction model developed in this study reduced HBS undiagnosed rate from 19.4% to 0% at the 35% decision threshold, providing a robust tool for developing personalized calcium supplementation strategies in clinical practice.

### 3.5 Interpretability analysis

**Figure 5:**
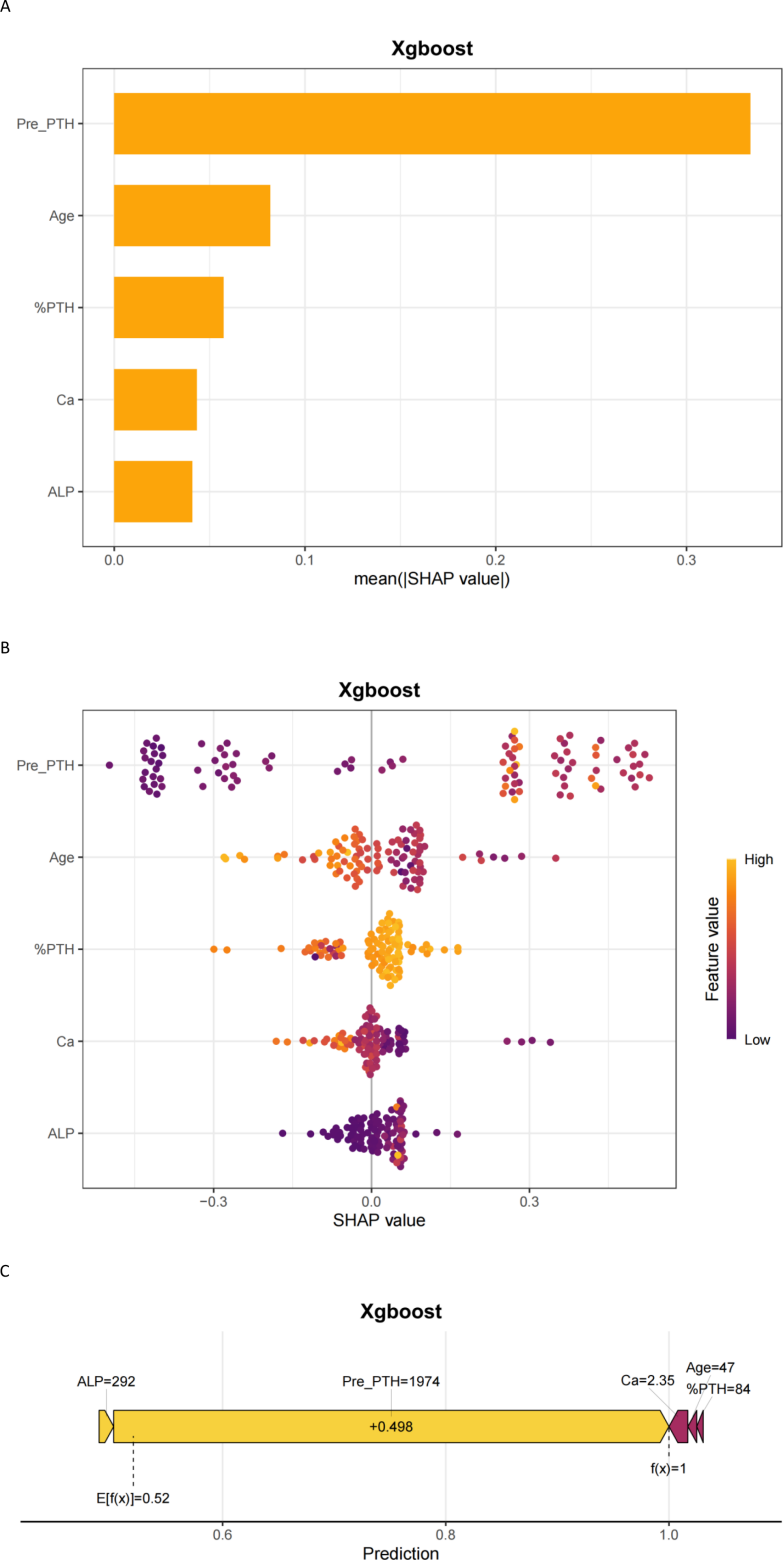
Interpretability of machine learning predictions using SHAP. **(A)** SHAP importance plot; **(B)** SHAP beeswarm plot; **(C)** SHAP force plot. Pre-PTH, preoperative parathyroid hormone; %PTH, [(Preoperative PTH – PTH at skin closure)/Preoperative PTH] × 100%; ALP, Alkaline phosphatase (U/L); Ca, Serum calcium (mmol/L).

The SHAP interpretability method system reveals the decision-making mechanism of XGBoost model in predicting HBS risk. **Figure 5A** (feature importance bar chart) shows that preoperative PTH level (mean SHAP = 0.32) is the most significant predictive factor, followed by Age,%PTH, Ca, and ALP. **Figure 5B** (bee swarm plot) further clarifies feature directionality: Preoperative high calcium levels significantly reduce HBS risk (SHAP value negative skew), while younger patients and elevated ALP drive risk increase (SHAP value positive clustering). The individualized decision mechanism is illustrated in **Figure 5C** (force plot): Taking a 47-year-old patient (preoperative PTH 1974 pg/mL, %PTH 84, calcium 2.35 mmol/L, ALP 292 U/L) as an example, the model predicts a 98.0% probability of HBS.

### 3.6 Application of model

To bridge the gap between predictive analytics and clinical utility, we operationalized the XGBoost model through an intuitive web-based platform ( *https://chaiyalin.shinyapps.io/make_web/* ). This tool enables clinicians to input patient-specific parameters—including preoperative PTH, ALP, serum calcium, age, %PTH—to generate real-time HBS risk stratification. The platform’s device-agnostic architecture ensures seamless access across desktop and mobile environments without requiring specialized software installation.

## 4 Discussion

Postoperative hungry bone syndrome (HBS) is a frequent complication following parathyroidectomy (PTX) in secondary hyperparathyroidism (SHPT) patients. We found that preoperative PTH was the most important risk factor for postoperative HBS. The possible reason is the rapid reduction in PTH levels shifts the bone turnover equilibrium toward mineralization, resulting in massive calcium influx into osteoid tissue and subsequent hypocalcemia (19, 20). This study identifies %PTH as independent predictors of HBS, leveraging PTH’s short half-life (3 – 5 minutes) to reflect real-time parathyroid functional dynamics.

Alkaline phosphatase (ALP), a biomarker of osteoblast activity, emerged as a critical predictor of HBS, consistent with prior studies (27–29). Elevated preoperative ALP levels signify heightened bone turnover, which exacerbates postoperative calcium sequestration (30). Notably, ALP demonstrates stronger correlations with bone histomorphometric changes and dialysis patient mortality than PTH (31), underscoring its clinical utility in risk stratification. While bone-specific ALP (B-ALP) offers superior specificity for bone metabolism (21), its exclusion from routine clinical assays in primary care settings limits its practical applicability. Our study corroborates prior evidences (23, 32) demonstrating that preoperative hypocalcemia independently predicts postoperative HBS, likely reflecting an elevated baseline bone remodeling state in SHPT patients (33). Mechanistically, abrupt postoperative PTH suppression disrupts the bone resorption-formation equilibrium, triggering accelerated osteoblast-driven mineralization and subsequent calcium efflux from circulation to bone tissue. Notably, younger age emerged as an independent HBS risk factor, aligning with findings by Kritmetapak et al. (34) and He et al. (23). This association may stem from heightened osteoblast activity and skeletal calcium utilization efficiency in younger individuals. Conversely, older patients exhibited reduced susceptibility, potentially attributable to age-related declines in 1α-hydroxylase activity, chronic malnutrition, and impaired osteoblast calcium uptake capacity. However, conflicting data from Gong et al. (35) associate advanced age with HBS risk, possibly due to prevalent vitamin D deficiency and protein-energy wasting in elderly populations. These discrepancies likely arise from cohort heterogeneity and surgical technique variations, underscoring the need for multicenter studies to clarify age-specific risk profiles. Additionally, we observed a reduced HBS rate among patients using bisphosphonates preoperatively, which aligns with other studies. Some authors have documented that bisphosphonates can improve HBS (19, 36, 37). Davenport et al. (19)described a study showing that pamidronate could decrease postoperative HBS rates.

In recent years, machine learning has shown great potential in disease diagnosis and prognosis. However, few studies have been conducted to predict postoperative HBS using ML models. This study introduces an interpretable machine learning model that individualizes HBS risk prediction using five clinically accessible variables. The model’s operationalization as a web-based calculator enables real-time risk stratification, aligning with KDIGO guidelines for proactive postoperative management. Internal validation via cohort partitioning demonstrated robust discriminatory performance, supporting its reliability for clinical deployment. Moreover, traditional evaluation methods often exhibit high rates of missed diagnoses. Our XGBoost model effectively reduces diagnostic gaps by integrating multidimensional features such as%PTH levels while maintaining appropriate specificity. We have further developed a risk stratification framework (low, medium, and high-risk) based on this threshold, establishing corresponding preventive measures and monitoring strategies. This model is designed for intraoperative decision support, enabling surgeons to adjust calcium prophylaxis strategies based on real-time %PTH dynamics. High-risk patients trigger immediate IV calcium infusion protocols.

However, several limitations warrant consideration. This study has the following limitations: 1. The single-center retrospective design limits model generalizability, requiring multicenter prospective validation; 2. The HBS definition is based solely on biochemical indicators without incorporating symptom assessment, which may lead to minor misclassification; 3. Key bone metabolism markers such as bone-specific alkaline phosphatase and osteocalcin are absent (38); 4. Model calibration performance may drift over time, necessitating periodic recalibration; 5. Differences in surgical techniques across centers and variations in surgeons’ expertise may affect model applicability.

## 5 Conclusion

Our findings demonstrate that %PTH exhibits a strong independent association with postoperative hungry bone syndrome (HBS). Leveraging machine learning algorithms, we developed and validated a predictive model incorporating five clinically accessible preoperative parameters: 1) preoperative PTH, 2) %PTH, 3) age, 4) serum calcium, and 5) alkaline phosphatase (ALP). This model serves as a practical clinical tool for early identification of high-risk patients, enabling targeted prophylactic interventions such as preoperative calcium optimization and intensified postoperative monitoring. By utilizing routinely available preoperative biomarkers, our approach bridges the gap between predictive analytics and actionable clinical decision-making in CKD-associated SHPT management.

## Data Availability

The raw data supporting the conclusions of this article will be made available by the authors, without undue reservation.
