# Supplementary material for: Machine learning-based predictive model for hungry bone syndrome following parathyroidectomy in secondary hyperparathyroidism
Source: Front Endocrinol (Lausanne). 2025 Sep 5;16:1635451. doi: 10.3389/fendo.2025.1635451 (PMC12446021; doi:10.3389/fendo.2025.1635451)
Supplement: Supplementary file 1 [file Table1.docx]

Supplementary Table1. Distribution of Missing Data.

**Supplementary Table1.** **Distribution of Missing Data.**

| Variable Names | Percentage of missing data, % |
| --- | --- |
| Wt(kg) | 1.7 |
| BMI(kg/m2) | 1.7 |
| Dialysis time(years) | 1.1 |
| CYSC(mg/L) | 10.5 |
| UA(µmol/L) | 3.9 |
| ALP（U/L） | 5.5 |
| ALB(g/L) | 9.3 |
| TG(mmol/L) | 2.2 |
| TC(mmol/L) | 2.2 |
| HDL(mmol/L) | 2.2 |
| LDL(mmol/L) | 2.2 |
