# Supplementary material for: Machine learning-based predictive model for hungry bone syndrome following parathyroidectomy in secondary hyperparathyroidism
Source: Front Endocrinol (Lausanne). 2025 Sep 5;16:1635451. doi: 10.3389/fendo.2025.1635451 (PMC12446021; doi:10.3389/fendo.2025.1635451)
Supplement: Supplementary file 2 [file Table2.docx]

Supplementary Table2.The regression coefficients of the variables in the Lasso regression.

| var | coef |
| --- | --- |
| (Intercept) | 1.54963041574123 |
| Ca | -0.047074519 |
| ALP | 2.31694599045583e-06 |
| Pre_PTH | 0.000181768599405898 |
| %PTH | 0.000653229624026999 |
| Age | -0.004863199 |
