# Supplementary material for: Machine learning-based predictive model for hungry bone syndrome following parathyroidectomy in secondary hyperparathyroidism
Source: Front Endocrinol (Lausanne). 2025 Sep 5;16:1635451. doi: 10.3389/fendo.2025.1635451 (PMC12446021; doi:10.3389/fendo.2025.1635451)
Supplement: Supplementary file 3 [file Table3.docx]

Supplementary Table3.The regression coefficients of the variables in the Lasso regression.

| Model | Models including %PTH | Model excluding %PTH |
| --- | --- | --- |
| Logistic | 0.876,95%CI:0.776−0.971 | 0.870,95%CI:0.777−0.943 |
| SVM | 0.865,95%CI:0.766−0.964 | 0.861,95%CI:0.747−0.941 |
| NeuralNetwork | 0.833,95%CI:0.718−0.947 | 0.839,95%CI:0.723−0.946 |
| Xgboost | 0.878,95%CI:0.779−0.973 | 0.869,95%CI:0.770−0.959 |
| KNN | 0.869,95%CI:0.762−0.976 | 0.809,95%CI:0.708−0.911 |
| Adaboost | 0.821,95%CI:0.708−0.915 | 0.804,95%CI:0.692−0.917 |
| CatBoost | 0.874,95%CI:0.765−0.983 | 0.871,95%CI:0.760−0.974 |
