# Supplementary material for: Machine learning-based predictive model for hungry bone syndrome following parathyroidectomy in secondary hyperparathyroidism
Source: Front Endocrinol (Lausanne). 2025 Sep 5;16:1635451. doi: 10.3389/fendo.2025.1635451 (PMC12446021; doi:10.3389/fendo.2025.1635451)
Supplement: Supplementary file 4 [file Table4.docx]

Supplementary Table4.The regression coefficients of the variables in the Lasso regression.

| Model | Youden_Threshold | Youden_Sensitivity | Youden_Specificity | DCA_Optimal_Threshold | Max_Net_Benefit | Net_Benefit_at_35 |
| --- | --- | --- | --- | --- | --- | --- |
| Logistic | 0.367690590250542 | 0.774193548387097 | 0.909090909090909 | 0.01 | 0.580712788259958 | 0.431059506531205 |
| SVM | 0.157813686333001 | 0.870967741935484 | 0.772727272727273 | 0.01 | 0.580712788259958 | 0.384615384615385 |
| NeuralNetwork | 0.392792042575361 | 0.774193548387097 | 0.863636363636364 | 0.01 | 0.580712788259958 | 0.420899854862119 |
| Xgboost | 0.48859891295433 | 0.870967741935484 | 0.818181818181818 | 0.01 | 0.580712788259958 | 0.361393323657475 |
| KNN | 0.41105873204672 | 0.806451612903226 | 0.863636363636364 | 0.01 | 0.562988374309129 | 0.420899854862119 |
| Adaboost | 0.208187571459695 | 0.806451612903226 | 0.681818181818182 | 0.01 | 0.470364017533829 | 0.400580551523948 |
| CatBoost | 0.575266956240613 | 0.903225806451613 | 0.772727272727273 | 0.01 | 0.580712788259958 | 0.361393323657475 |
