# Supplementary material for: Machine learning-based predictive model for hungry bone syndrome following parathyroidectomy in secondary hyperparathyroidism
Source: Front Endocrinol (Lausanne). 2025 Sep 5;16:1635451. doi: 10.3389/fendo.2025.1635451 (PMC12446021; doi:10.3389/fendo.2025.1635451)
Supplement: Supplementary file 5 [file Table5.docx]

Supplementary Table5.

| Model | Brier_Score |
| --- | --- |
| Logistic | 0.1558 |
| SVM | 0.1861 |
| NeuralNetwork | 0.1839 |
| Xgboost | 0.1776 |
| KNN | 0.1728 |
| Adaboost | 0.1813 |
| CatBoost | 0.1919 |
